# Study protocol for a randomized controlled trial of the Parent–Child Assistance Program: a case management and home visiting program for people using substances during pregnancy

**DOI:** 10.1186/s13063-024-08098-6

**Published:** 2024-04-16

**Authors:** Erin J. Maher, Susan A. Stoner, Julie Gerlinger, A. C. Ferraro, Heather Lepper-Pappan

**Affiliations:** 1 Department of Sociology, University of Oklahoma, 780 Van Vleet Oval, Kaufman Hall 311, Norman, OK 73019 USA; 2 Department of Psychiatry & Behavioral Sciences, Addictions, Drug & Alcohol Institute, University of Washington School of Medicine, 1959 NE Pacific Street, Box 356560, Seattle, WA 98195-6560 USA

**Keywords:** Pregnancy, Early childhood, Home visiting, Case management, Substance use disorders, Randomized controlled trial, Recovery support services

## Abstract

**Background:**

Perinatal substance use can have significant adverse effects on maternal and child health and family stability. Few interventions are specifically designed to address this significant public health problem. The Parent–Child Assistance Program (PCAP) is a 3-year case management and home-visiting intervention that seeks to help birthing persons with at-risk substance use during pregnancy to achieve and maintain substance use disorder recovery and avoid exposing future children to substances prenatally. At-risk refers to a level of substance use that creates problems in the individuals’ lives or puts them or their children at risk of harm either prenatally or postnatally. Although the program has consistently shown substantial pre- to post-intervention improvements in its participants, PCAP remains to be tested with a rigorous randomized controlled trial (RCT). This study protocol describes a randomized controlled trial that aims to examine the effectiveness of the intervention compared to services as usual in affecting primary outcomes related to substance use and family planning. Secondary outcomes will concern connection to recovery support services and family preservation.

**Methods:**

Using an intent-to-treat design, the study will recruit from two metro areas in Oklahoma and enroll 200 birthing individuals who are pregnant or up to 24 months postpartum with at-risk substance use during their current or most recent pregnancy. Participants will be randomly assigned, stratified by location, to receive either PCAP or services as usual for 3 years. Participants in the PCAP condition will meet with their case manager approximately biweekly over the course of the intervention period, in their local communities or in their own homes whenever possible. Case managers will assist with goal setting and provide practical assistance in support of participants’ goals. Primary and secondary outcomes will be assessed at baseline and 12, 24, and 36 months post-baseline using the Addiction Severity Index interview and a self-administered survey.

**Discussion:**

Results from this trial will help to gauge the effectiveness of PCAP in improving parent and child well-being. Results will be reviewed by federal clearinghouses on home-visiting and foster care prevention to determine the strength of evidence of effectiveness with implications for federal financing of this program model at the state level.

**Trial registration:**

ClinicalTrials.gov NCT05534568. Registered on 6/8/2022.

**Supplementary Information:**

The online version contains supplementary material available at 10.1186/s13063-024-08098-6.

## Background {6a}

### Prevalence of perinatal substance use

Perinatal substance use is a significant public health problem associated with adverse maternal [1, 2] and neonatal [3, 4] outcomes. According to the 2022 National Survey on Drug Use and Health, 5.3% of pregnant women reported past month binge alcohol use and 9.6% reported past month illicit drug use [5]. In a 2019 population-based sample of postpartum women, one in 15 (6.6%) self-reported using prescription opioid pain relievers during pregnancy, and of those, one in five indicated misuse [6]. Rates of substance use disorders (SUDs) among pregnant and parenting women are concerningly high, particularly among low-income women. One study examining three state Medicaid programs from 2013 to 2016 found 11.3% of postpartum women had a SUD [7], and an examination of delivery hospitalizations between 2000 and 2018 revealed that the prevalence of SUD diagnoses has been increasing [1]. Among those who achieved abstinence in the last month of pregnancy, rates of relapse can be as high as 80% in the 2 years after delivery [8].

### Poor outcomes from perinatal substance use

Perinatal substance use can have significant adverse effects on maternal and child health. Drug overdose is now a leading cause of death during and shortly after pregnancy. Overdose deaths among pregnant and postpartum individuals increased by 81% from 2017 to 2020 [9], and up to 22% of pregnancy-associated deaths are attributable to substance use [10]. Substance use during pregnancy is linked to decreased prenatal and postpartum care access [11], and the use of opioids or illicit stimulants during pregnancy is associated with a fourfold increase in risk of postpartum readmission [2]. Women with SUDs have a 6.2-fold increased risk of postpartum suicide attempts, and the risk nearly doubles for those with both SUD and psychiatric diagnoses [12].

### Poor outcomes for children

Alcohol use during pregnancy is a leading cause of preventable birth defects [13] and results in fetal alcohol spectrum disorders with lifelong impacts [14] and high costs of care [15]. Prenatal exposure to cannabis, opioids, and stimulants has also been found to have adverse effects on newborns. Cannabis is often believed to be harmless or even beneficial by those who use it during pregnancy [16], yet such use has been linked to lower birthweight, decreased length, and smaller head circumference [17]. Prenatal opioid use is associated with maternal mortality, stillbirth, preterm birth, low birthweight for gestational age, neonatal abstinence syndrome, need for neonatal intensive care, and prolonged hospital stays [18, 19]. Prenatal stimulant use is linked to fetal growth restriction, preterm birth, and low birthweight for gestational age [20, 21].

### Need for effective interventions

Given the scope and impact of perinatal substance use on maternal and child health outcomes, effective interventions to address this problem are sorely needed. Currently under the federal Maternal, Infant, and Early Childhood Home Visiting (MIECHV) program’s clearinghouse—the Home Visiting Evidence for Effectiveness Clearinghouse (HomeVEE)—no programs with strong evidence of effectiveness are targeted specifically to pregnant people or parents with SUDs. Moreover, the number of HomeVEE-rated programs that meet criteria for strong evidence and produce statistically significant results on parental SUDs is meager and limited in scope. At the time of this writing, only Family Spirit [22], Nurse Family Partnership [23], and Healthy Families America [24] have documented significant effects on parental SUD. Only two programs are rated as highly effective for addressing parental SUD to prevent foster care placements under the new clearinghouse for reviewing foster care prevention programs established by the Family First Prevention Services Act of 2018 (FFPSA) (42 USC § 675a).

In this article, we describe a study protocol to evaluate a program specifically designed to address perinatal substance use. The Parent–Child Assistance Program (PCAP) is a 3-year case management and home-visiting intervention for pregnant and postpartum individuals with at-risk substance use during their most recent pregnancy, that is, a level of substance use that creates problems in the individuals’ lives or puts them or their children at risk of harm either prenatally or postnatally. Although individuals completing the program have consistently shown significant pre- to post-intervention improvements, PCAP remains to be tested with a rigorous randomized controlled trial (RCT), often a requirement to be considered an evidence-based program under federal research standards [25, 26]. Therefore, we are conducting a RCT of PCAP within the state of Oklahoma. In state fiscal year 2020, 1,024 newborns who were tested were substance exposed, justifying the target sample size and the need for this project in Oklahoma [27].

## Research design and methods

### Research objectives {7}

The purpose of this RCT is to evaluate the impacts of PCAP on parental recovery, connection to recovery support services, family well-being, and the likelihood of future substance-exposed births. Consistent with our focus on prevention of prenatal substance exposure, this trial focuses on pregnant and postpartum birthing individuals, which includes people with different gender identities who are capable of becoming pregnant.^1^

We hypothesize that individuals receiving the intervention will experience better outcomes than those receiving services as usual in the community over the same 3-year period. Primary outcomes include those related to SUD recovery and the prevention of substance-exposed births. Secondary outcomes include connection to recovery support services, parental custody, criminal justice involvement, and financial stability. The study protocol has been approved by the University of Oklahoma Institutional Review Board, which is responsible for ongoing oversight, the Oklahoma Department of Mental Health and Substance Abuse Services, and the Oklahoma Department of Human Services. The project has received a Certificate of Confidentiality from the National Institutes of Health and is registered on ClinicalTrials.gov.

### Study design {8} {9} {14}

We will evaluate PCAP using a RCT with an intent-to-treat design in two sites in Oklahoma: Oklahoma City and Tulsa, including their surrounding areas. The study will recruit and enroll 200 participants, with 50 control and 50 treatment group participants at each site. Both groups will participate in data collection at baseline and at 12, 24, and 36 months post-baseline, with 36 months post-baseline being the primary endpoint. Study participants will not be constrained from accessing any other services or interventions that may be available in their community.

### Description of the intervention {11a} {11b} {11d}

The Parent–Child Assistance Program (PCAP) has been described in detail elsewhere [25, 28]. In brief, PCAP is a 3-year intervention for pregnant and post-partum individuals with problematic or at-risk substance use during their most recent pregnancy. Built on the theoretical foundations of relational theory [29], the transtheoretical model of behavior change [30], and harm reduction [31], PCAP aims to support birthing individuals in completing SUD treatment and link them with comprehensive community resources to help them stay in recovery, build healthy lives, and avoid exposing any future children to alcohol or drugs prenatally. PCAP provides program participants with a bachelor’s-level case manager (CM), with whom they meet approximately bi-weekly, in participants’ own homes or elsewhere in the community. CMs typically work with up to 20 participants and their families, serve as role models, help them to articulate their own goals and take steps to reach them, and provide transportation and practical assistance when needed in support of participants’ goals. The intervention manual is available at https://pcapwa.org/manual. There are no formal criteria for modifying the intervention for a given participant. The design of the intervention provides for flexibility to adjust the frequency of contacts according to the participant’s needs and preferences while aiming for an average of two face-to-face contacts per month over the course of the intervention. Consistent with the intent-to-treat approach, participants who ask to suspend their participation in the intervention or withdraw from the intervention completely will remain in the study unless they specifically ask to withdraw from the study as a whole.

### Description of the control condition {6b}

Participants randomly assigned to the control condition will not receive PCAP but will not be constrained from accessing available services as usual in their community. They will be provided with a list of local services and resources. These resources include information for accessing substance use disorder and mental health treatment and support, health care, family planning, domestic violence services, housing and food assistance, and legal services.

### Participants and recruitment {15}

The recruitment strategy will leverage a database of service providers that includes SUD treatment facilities, hospitals, birthing centers, judicial personnel, tribal groups, Indian health centers, mental health providers, and other service providers and agencies that may encounter the target population, such as WIC/SNAP/food stamp/welfare offices, emergency rooms/urgent care, homeless shelters, parenting classes, health departments, domestic violence shelters, community health centers (taking Medicaid), Planned Parenthood, and hospital social workers. The database is compiled by research and direct service staff based on their knowledge of service providers in the communities. Recruitment also occurs through public webinars, presentations and vendor tables at local and state conferences and symposia, presentations at local organizations, media attention, flyers, and social media.

### Screening, consent, enrollment {10} {26a} {26b}

Prospective participants may self-refer or be referred by friends, family, or service providers via phone or by completing a referral form on the project Web page. A brief screening conducted by research staff ascertains eligibility. Inclusion criteria are as follows: (1) being pregnant or up to 24 months postpartum, (2) self-reporting at-risk alcohol or drug use during the most recent pregnancy, and (3) lacking adequate connection to community/social/health service providers. Alternatively, consistent with the PCAP model, individuals are eligible if they have a child (of any age) with Fetal Alcohol Spectrum Disorder, continue to use alcohol, and are in their childbearing years. Exclusion criteria include not being able to communicate in English, being under the age 18 years, having a significant cognitive or mental health condition that would preclude the ability to consent to be in the study, and being enrolled in a program that provides duplicative services. Informed consent will be obtained from eligible individuals by the Research Coordinator before administering the baseline data collection instruments. Randomization will occur upon completion of the baseline data collection.

#### Assessment procedures

Whenever feasible, participants will complete study assessments at a private workstation at one of the study sites, connecting with the research coordinator via Zoom videoconferencing. In situations where a participant cannot come to the site, assessments may be conducted with the interview portion via phone and the survey portion completed via web link or on paper forms to be returned via US Mail to the research office. Alerts will be used to remind research staff of data collection intervals. To maintain currency of contact information, research staff will check in with study participants in both groups every 3 months between data collection intervals with a brief online form sent by text message.

### Randomization procedures {16a} {16b} {16c}

**Fig. 1:**
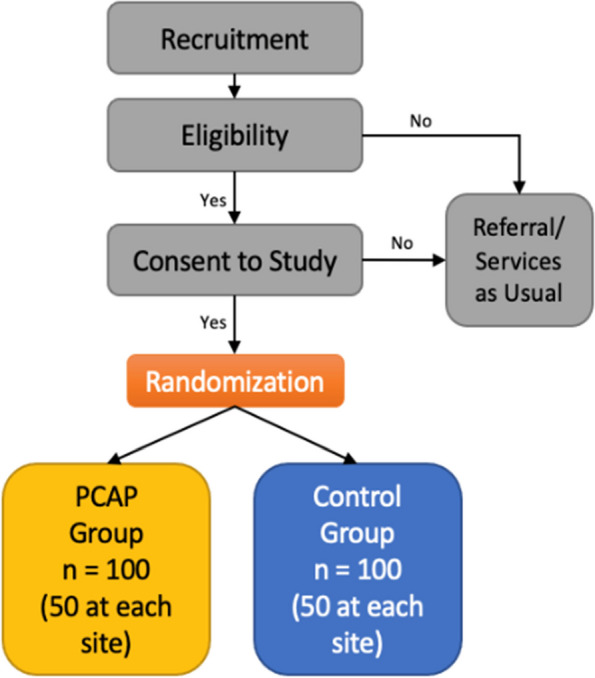
Randomization and enrollment process

Group assignment will occur via an online random assignment generator called Research Randomizer [32], which relies on a computer algorithm for randomization within REDCap, the data management system described below. Randomization will be stratified by location to produce equal numbers of participants in the treatment and control groups at each site. Neither the research staff using the system nor the participant will know the group assignment before the baseline data are collected. Figure 1 displays enrollment and randomization processes.

### Blinding {17a} {17b}

Group membership will not be blinded after assignment. Participants and staff will be aware of whether a participant is assigned to the treatment or control group by necessity given the nature of the intervention.

### Post trial care {30}

Participants will continue with services as usual post-trial.

### Measures {12} {20a}

For this study, primary outcomes concern parental SUD recovery and prevention of future substance-exposed births. Specifically, we will assess substance use (recency and frequency), SUD treatment completion, duration of abstinence from substance use, use of family planning methods, and number of subsequent substance-exposed births. Secondary outcomes concern parent–child linkage to recovery supports and social services, prevention of foster care placement, parent–child reunification, and family stability. Recovery supports/social services include housing, health care, family planning, mental health treatment, support groups, food assistance, daycare/childcare, and legal services. Prevention of foster care placement and parent–child reunification will be examined in terms of the living arrangement and legal custody of the index child and the number of months the child lived with the participant. Family stability refers to parental education and employment status, stable housing, and no new involvement with the criminal justice system.

### Data management {19}

Whenever possible, data will be directly entered into REDCap, a HIPAA-compliant data management platform. During the interview portion of the assessment, the research coordinator will enter participant responses into REDCap as they are given. When the self-administered survey is completed online, data will be stored as each question is answered. We anticipate that participants’ completing the self-administered survey on paper will be rare. When that happens, the paper forms will be stored in a locked cabinet until the data are entered by the research coordinator and data entry accuracy is verified by another study staff member, after which the paper forms will be shredded (Table 1).

**Table 1:** Measurement instruments, constructs, and purpose {18a}

Instrument	Constructs	Measurement purpose
Addiction Severity Index	Demographics, alcohol and drug use, family planning methods, legal involvement, medical conditions, psychiatric disorders, employment, connection to community resources, family history, child custody adverse childhood experiences	Primary and secondary outcomes
Brief Version of Addiction Severity Index	Current alcohol and drug use, family planning, legal involvement, mental status, psychiatric disorders, employment, connection to community resources, child custody	Primary and secondary outcomes
Self-administered survey	Criminal justice involvement, expanded adverse childhood experiences (baseline only), social support, maternal attachment, post-traumatic stress, psychological distress, self-esteem, self-efficacy, addiction beliefs, positive parenting, knowledge of child development, benevolent child experiences, working alliance inventory	Secondary outcomes and mediators

The 5^th^ edition of the Addiction Severity Index (ASI) is a semi-structured interview instrument that assesses the following domains: medical status, employment/support status, substance use, legal status, family history, family/social relationships, and psychiatric status [33]. This instrument has been supplemented with additional items developed by subject matter experts, straightforwardly worded. These include items to assess drug use during pregnancy, reproductive history, and adverse life experiences not assessed in the ASI. Substances assessed include and expand upon those in the ASI: alcohol, heroin, fentanyl, methadone, other opiates/analgesics, barbiturates, sedatives/tranquilizers, cocaine, methamphetamine, other amphetamines, cannabis, hallucinogens, inhalants, and other substances. Additional items assess birth control use, number and living situation of biological children, adverse life experiences, and child protective services involvement. Items have also been created to assess the services participants have received in the preceding year, including healthcare for mother and child(ren), family planning, mental health, housing, emergency funds, food/clothing/supplies, legal aid, domestic violence, childcare, support groups, public schools, and public health nursing. Each service is coded as to whether the participant expressed a need for that service (yes = 1/no = 0) and whether the service was received (yes = 1/no = 0). An abbreviated (brief) version of the ASI that omits time invariant/historical information is administered at the annual follow-up assessments.

#### Self-administered survey (SAS)

**Fig. 2:**
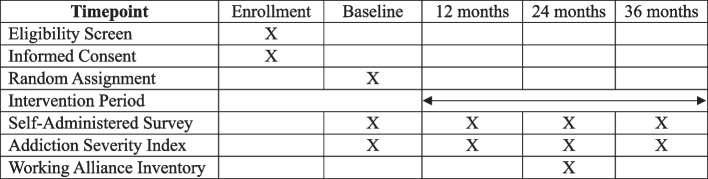
Schedule of enrollment, intervention, and assessments {13}

The SAS was developed by the investigator team, drawing primarily upon standardized scales and measures used in addictions research and related subfields that have solid psychometric properties. The SAS elicits information from participants along several dimensions covering a wide range of participant behavior metrics and social psychological concepts important for addiction research, including criminal justice involvement, additional adverse childhood experiences (ACEs) from an expanded ACEs instrument [34], social support [35], maternal attachment (antenatal [36] and postnatal [37]), mental health (PTSD [38] and psychological distress [39]), self-esteem [40] and self-efficacy [41], addiction beliefs [42, 43], parenting practices [44], and parent/child experiences [45] and child development [46]. These measures allow us to examine both predictors of success, as well as additional secondary outcomes stemming from the intervention. Figure 2 presents the schedule of enrollment, intervention, and assessments.

### Intervention adherence {11c} {18b}

Adherence to the intervention on the part of the participant is defined as engagement with the CM. Strategies to improve adherence will include (1) requiring the CMs to reach out proactively to participants in the intervention condition to arrange biweekly face-to-face meetings, (2) offering transportation to participants in support of their goals, (3) helping participants to access tangible goods (e.g., diapers, food assistance) in support of family needs, and (4) obtaining consent to contact a friend or family member in the event the participant cannot be reached/located.

#### Fidelity monitoring

Fidelity to the PCAP model will be monitored by examining time logs for CMs and monthly and semi-annual reports of participant status. CMs will record the amount of time spent working directly with or on behalf of each participant, including face-to-face meetings, time spent transporting the participant, phone communication with the participant, and other time spent working on a participant’s case without the participant present, such as working with an agency on behalf of the participant, time spent with participant’s family, and time spent corresponding with the participant. Specifically, we will monitor the number of visits per month, maintaining an expectation that CMs meet with each intervention participant twice per month and that CMs spend at least 55% of their time working with or on behalf of intervention participants. A case management supervisor will monitor time spent with participants and intervention milestones, providing feedback when deviations from fidelity are noted.

### Data analysis plan {20b} {20c}

After checking for equivalency between groups on key baseline measures, we will conduct cross-sectional, bivariate analyses to assess differences between treatment and control groups at 12-, 24-, and 36-month timepoints. Evaluation of effectiveness will utilize both intent-to-treat (ITT) and as-treated (AT) approaches [47]. In the ITT analysis, all randomly assigned participants will be included in the analysis, regardless of the extent to which they received the intervention. In the AT analysis, only those who remain engaged in the intervention at the timepoint of interest will be included. Additionally, we will examine the dosage of the intervention and intervention site as moderators of between-group effects. Other potential moderators to be examined include participant race/ethnicity, marital status, age, and criminal justice involvement.

We anticipate that missing data will not be missing completely at random and are unlikely to be missing at random due to the nature of SUD research. For example, participants with more severe SUDs may be more likely to die from overdose and thus contribute to missing data. We will acknowledge this limitation when the findings are disseminated. Bivariate analyses will use pairwise deletion in the case of missing data. Regression analysis examining mediation and moderation will use listwise deletion. Statistical modeling will use full information maximum likelihood estimation whenever possible.

#### Power and sample size

The sample size of 200 participants will provide power of .80 to detect at least small-to-medium effects (Cohen’s *d* = .40) using two-tailed, between-groups, planned comparisons with an error probability of .05, reflecting the ceiling of statistical power. Given the nature of SUDs and their potential impact on the stability of participants’ lives, we conservatively estimate that 33% will attrit from the study before the primary endpoint of 36 months. This is more conservative than what has been observed in Washington State, where 72% of participants who enrolled in PCAP remained in the program for 36 months. A sample size of 132 participants will provide power of .80 to detect at least medium effects (Cohen’s *d* = .50), reflecting the anticipated floor of statistical power.

### Data and safety monitoring {21a} {21b} {22} {24}

All procedures will be reviewed and approved by the University of Oklahoma IRB with annual reviews. Exclusively using psychosocial methods, the intervention to be evaluated in this study is deemed low risk. Accordingly, an external data and safety monitoring board will not be used. The study PIs (EM, SS) will be jointly responsible for data and safety monitoring and will meet for this purpose at least semi-annually. Interim analyses will be conducted at least every 6 months. Every week, the investigators review recruitment and retention rates. Semi-annually, they review baseline demographics, adverse events, and primary and secondary outcomes and consider whether there are relatively greater risks in either arm of the trial that warrant stopping the trial. Anticipated potential and reportable adverse events include but are not limited to breaches of confidentiality, death of a subject, reports of acts of violence, child abuse, and complaints about the study or the research team. Per state law, child abuse will be reported to Children’s Protective Services within 48 h, as stated in the consent form. Should an unanticipated adverse event occur, the following steps will be taken: (1) the participant will be informed as to the nature of the event, and its possible implications; (2) the PIs will immediately notify the IRB; (3) the PIs will consult with the Co-Is to review existing safeguards to determine how and why the event occurred; (4) data collection will be halted until new/modified safeguards are developed and implemented; (5) the IRB will review the new procedures, and data collection will resume after a new approval is granted. In addition, the funding agencies will be informed of any actions taken by the IRB at any time as a result of continuing reviews.

### Frequency and plans for auditing trial {23}

The trial is overseen by a group of investigators and a full-time Project Director at the University of Oklahoma and the University of Washington. A full-time research coordinator is responsible for data collection. This group oversees the implementation and evaluation of PCAP. The direct services are managed by a Supervisory/Manager in OU Outreach’s Southwest Prevention Center (SWPC). The Project Director meets weekly with the research coordinator and the investigator team, along with other meetings as needed. The investigator team and Project Director meet monthly with the SWPC to coordinate activities. The Direct Services team meets weekly with the SWPC Manager. The investigators meet weekly to closely monitor the PCAP trial and discuss the progress of the study and any issues that may arise during its conduct. Given the low-risk nature of the intervention, no external monitoring committee is used. The project also has a Parent Advisory Committee with their own leadership structure who meet quarterly with the full project teams. The Parent Advisory Committee also is invited to monthly investigator team meetings. They have advised on measurement, recruitment, retention, hiring, and direct services. They have also attended conferences and represented PCAP in public forums. We have no plans for an independent monitor.

### Protocol amendments {25}

Since this project employs continuous quality improvement procedures, modifications to data collection forms or documented procedures are submitted to the IRB of record. Amendments to the study protocol will only be made if deemed essential in the face of emergent problems according to the study PIs. If the IRB deems it necessary, previously enrolled participants will be informed and re-consented. All modifications are documented in our research and direct service guides. Any deviations to the protocol or adverse events will be reported to the IRB. Sponsors and funders are notified of any significant changes in our regular progress reporting. Major protocol modifications will be uploaded to the clinical trial registry.

### Confidentiality {27}

Participants will sign a HIPAA waiver to allow their protected health information to be maintained and used by the study. As noted, data will be managed with a HIPAA-compliant platform (REDCap) and stored on a secure, HIPAA- and HITECH-compliant server at the University of Oklahoma Health Sciences Center. Direct identifiers will be destroyed upon completion of the trial or as dictated by the IRB. In accordance with HIPAA and 42 CFR Part 2, participants’ written consent will be required to identify their connection to the trial, which will have a federal certificate of confidentiality from the National Institutes of Health.

### Dissemination {31a} {31b} {31c}

Access to de-identified data will be provided upon reasonable request, including to the study sponsors, with execution of a formal data use agreement. Findings will be published regardless of whether study hypotheses are supported. Trial results will be disseminated via ClinicalTrials.gov, publications in peer-reviewed journals, presentations at national scientific meetings, presentations to the community, summaries on the study website, and reports to study sponsors, federal clearinghouses, and state policy makers. Authorship eligibility will be restricted to those who have made substantive contributions to conception or design or conduct of the study and who have contributed to the preparation of the work. All authors must be accountable for the work and approve of the final version to be published.

## Discussion

This RCT fills a gap for impact studies on programs for pregnant and parenting people with problematic substance use during pregnancy. In the protocol, we outline our major goals for the evaluation of PCAP and the primary and secondary outcomes, which we hypothesize to be impacted by the intervention. One of the strengths and challenges of this trial is primary data collection on a highly vulnerable population. This vulnerability of the study population translates into experiences that lead to tenuous circumstances with high degrees of residential mobility, thus making it difficult for researchers to conduct field-based trials. As noted, we anticipate attrition during the 3-year study period. Previous studies have shown that a 3-year intervention study requires a lot of resources to keep participants engaged and located [25, 28]. It is because of these difficulties, in part, that this population has gone understudied and few evidence-based interventions exist. While the collection of primary data (vs. solely relying on administrative data) provides us with a depth of theory-driven information on study participants and will allow us to examine mechanisms that mediate the intervention’s effects, a limitation of this approach is the reliance on self-reported measures, including substance use. Our consent form allows for future linking of participant records with cross-sector administrative records to test the validity of this approach, as funding allows.

Ancillary goals of this RCT include getting rated by federal clearinghouses for evidence-based programs in the realms of home-visiting and foster care prevention. In this regard, it fits squarely within the context of evidence-based policy making, which incentivizes social programs to conduct impact evaluations [48]. In the realm of home visiting and child welfare, the federal government provides financial incentives for states to select and implement evidence-based programs. Most importantly, PCAP provides intensive services to a highly vulnerable population with the potential to improve outcomes for both parents and children. If successful, and if the program shows positive impacts on the outcomes of interest, this trial could lead to substantial spread and replication of PCAP and help address the crisis of perinatal substance misuse nationwide.

## Trial status

The study protocol version is 1.18 11/14/23, which involved minor changes to the wording of the recruitment flyer for simplicity and clarification regarding eligibility criteria. This study began enrolling participants in December 2022 and is expected to enroll participants until December 2024.

### Supplementary Information

**Supplementary Material 1.**

## Data Availability

We are happy to make any study materials (e.g., consent form and study instruments) available to other researchers upon request. For verification purposes, we can make de-identified data available, upon request, to the maximum extent that is legally allowed under the terms of our IRBs and data use agreements.

## Abbreviations

SUD: Substance use disorder
MIECHV: Maternal, Infant, and Early Childhood Home Visiting
HomeVEE: Home Visiting Evidence for Effectiveness Clearinghouse
FFPSA: Family First Prevention Services Act
PCAP: The Parent–Child Assistance Program
RCT: Randomized controlled trial
CM: Case manager
ITT: Intent-to-treat
AT: As-treated
PI: Principal investigator
Co-I: Co-investigator
IRB: Institutional review board
HIPAA: Health Insurance Portability and Accountability Act
HITECH: Health Information Technology for Economic and Clinical Health
CFR: Code of Federal Regulations
REDCap: Research Electronic Data Capture (software)

## Footnotes

1 https://www.nih.gov/nih-style-guide/inclusive-gender-neutral-language
